# VitisCyc: a metabolic pathway knowledgebase for grapevine (*Vitis vinifera*)

**DOI:** 10.3389/fpls.2014.00644

**Published:** 2014-12-09

**Authors:** Sushma Naithani, Rajani Raja, Elijah N. Waddell, Justin Elser, Satyanarayana Gouthu, Laurent G. Deluc, Pankaj Jaiswal

**Affiliations:** ^1^Department of Botany and Plant Pathology, Oregon State UniversityCorvallis, OR, USA; ^2^Department of Horticulture, Oregon State UniversityCorvallis, OR, USA

**Keywords:** grape, VitisCyc, grapevine pathway database, *Vitis vinifera*, microarray

## Abstract

We have developed VitisCyc, a grapevine-specific metabolic pathway database that allows researchers to (i) search and browse the database for its various components such as metabolic pathways, reactions, compounds, genes and proteins, (ii) compare grapevine metabolic networks with other publicly available plant metabolic networks, and (iii) upload, visualize and analyze high-throughput data such as transcriptomes, proteomes, metabolomes etc. using OMICs-Viewer tool. VitisCyc is based on the genome sequence of the nearly homozygous genotype PN40024 of *Vitis vinifera* “Pinot Noir” cultivar with 12X v1 annotations and was built on BioCyc platform using Pathway Tools software and MetaCyc reference database. Furthermore, VitisCyc was enriched for plant-specific pathways and grape-specific metabolites, reactions and pathways. Currently VitisCyc harbors 68 super pathways, 362 biosynthesis pathways, 118 catabolic pathways, 5 detoxification pathways, 36 energy related pathways and 6 transport pathways, 10,908 enzymes, 2912 enzymatic reactions, 31 transport reactions and 2024 compounds. VitisCyc, as a community resource, can aid in the discovery of candidate genes and pathways that are regulated during plant growth and development, and in response to biotic and abiotic stress signals generated from a plant's immediate environment. VitisCyc version 3.18 is available online at http://pathways.cgrb.oregonstate.edu.

## Introduction

Grapevine (*Vitis* spp) is one of the most economically important and widely cultivated fruit crops in the world, covering ~8 million hectares and producing ~67.5 million tons of berries (http://www.oiv.int/). The majority of grape berries are processed into wine, but significant portions of the harvest provide fresh table grapes, raisins, juice, vinegar, and distilled spirits (http://faostat.fao.org/). In addition to its economic importance, grapes are rich in nutrients, metabolites, vitamins, volatile compounds, and dietary fibers—all of which offer potential benefits to human health (De et al., 2010; Smoliga et al., 2011; Wu and Hsieh, 2011).

The large-scale genomic datasets are of tremendous value in extending the knowledge of grape metabolism, scoring cultivar specific differences, and understanding how the plant's overall metabolism is affected by diseases and abiotic stress conditions resulting from changes in the photoperiod, temperature, moisture, climate etc. The current availability of genomics reasources for grape include genome sequence of two genotypes PN40024 and ENTAV 115 of the *Vitis vinifera* “Pinot Noir” cultivar (Jaillon et al., 2007; Velasco et al., 2007), and Vitis9kSNP array based genome diversity data for 950 *V. vinifera* and 59 *V. sylvestris* accessions (Myles et al., 2011). In addition, more than 50 varieties of grapevine are being sequenced at least at 10X coverage (http://www.vitaceae.org/index.php/Current_Sequencing_Projects) to probe the genetic diversity in the cultivated and wild grape accessions. Genetic linkage maps are available for various cultivars of *V. vinifera* (Doligez et al., 2002; Vezzulli et al., 2008) and quantitative trait loci (QTLs) have been identified for several traits including seedlessness, fungal disease resistance, and fruit yield components (Doligez et al., 2002; Fischer et al., 2004; Costantini et al., 2008; Salmaso et al., 2008). In addition to ESTs (Da Silva et al., 2005), microarrays and RNA-Seq data sets (Terrier et al., 2005; Waters et al., 2005, 2006; Espinoza et al., 2006; Cramer et al., 2007; Deluc et al., 2007, 2009; Fernandez et al., 2007; Grimplet et al., 2007; Pilati et al., 2007; Tattersall et al., 2007; Chervin et al., 2008; Figueiredo et al., 2008; Gatto et al., 2008; Lund et al., 2008; Mathiason et al., 2009; Zamboni et al., 2010; Zenoni et al., 2010), a few metabolomes and proteomes of grape berry have been published (Sarry et al., 2004; Giribaldi et al., 2007; Martinez-Esteso et al., 2011; Dai et al., 2013; Martínez-Esteso et al., 2013).

The lack of a comprehensive, evidence-based, grapevine-specific cellular metabolic framework limits the analysis and full utilization of the genomic scale expression data. We created VitisCyc, a metabolic pathway database, based on the genome sequence of the *Vitis vinifera* “Pinot Noir” accession PN40024, using 12X v1 annotations, provided by Centro di Ricerca Interdipartimentale per le Biotechnologie Innovative (CRIBI) (http://www.cribi.unipd.it/). VitisCyc was built on widely used BioCyc platform (Karp and Caspi, 2011; Karp et al., 2013) and contains metabolic and transport pathways, enzymes, transporters, genes, metabolites, and small biomolecules that are either experimentally determined or predicted to be present within a grapevine cell. The database is supported by extensive manual curation, and it facilitates incremental integration of existing and new information.

Grapes have been analyzed extensively for secondary metabolites related to color, taste, aroma etc. using chemical, physiological, and metabolic approaches. Thus, it provides an excellent opportunity for evidence-based curation of secondary metabolic compounds, reactions and pathways. At present, our curation efforts in VitisCyc are heavily focused on the secondary metabolic pathways. The knowledge derived from grape could be of use for discovering similar pathways in other fleshy fruits and to lesser extent for enriching model plant pathway databases, such as AraCyc (*Arabidopsis thaliana*) (Zhang et al., 2005), MedicCyc (*Medicago truncatul*a) (Urbanczyk-Wochniak and Sumner, 2007), RiceCyc (*Oryza sativa, rice*) (Dharmawardhana et al., 2013), and MaizeCyc (*Zea mays*, maize) (Monaco et al., 2013) etc., which significantly lack curation of secondary metabolic pathways.

VitisCyc allows users to search or browse for pathways, reactions, enzymes, genes, and compounds as well as supports analysis of user-defined, large-scale expression data in context of the overall cellular metabolic network using the OMICs-Viewer tool. VitisCyc can be used for developing cell, tissue or organ specific metabolic models and/or simulations representing changes in the grapevine cellular metabolic networks under various treatments or environmental stresses. In this publication, we report the development and curation of the VitisCyc database version 3.18 and show utility of OMICs-Viewer tool by analyzing expression data from pulp tissue of grape berries representing four ripening stages.

## Materials and methods

### Construction of VitisCyc

#### Genome, proteins, and annotation methods

VitisCyc was constructed based on the genome sequence of grape cultivar “Pinot Noir” (genotype PN40024) with 12X v1 annotations provided by CRIBI (http://genomes.cribi.unipd.it/grape/) that represents 29,971 protein coding genes. We used the Pathways Tools software (Karp et al., 2002) to create VitisCyc by following the instructions of the software developers on the input data file contents and formats. Accordingly, PRODUCT-TYPE code “P” was assigned for a protein or a hypothetical ORF. Each gene/protein record includes mandatory attributes: NAME (gene/protein name), and FUNCTION (name of the enzyme), and recommended attributes: Locus ID or gene ID, Enzyme Commission (EC) number and Gene Ontology (GO) assignments. The optional attributes include SYNONYM or gene symbols, and COMMENT (a free text description). Only the FUNCTION, SYNONYM, EC number, DBLINK and GO attributes have multiple values. The older grape GeneIDs from the 8X (Jaillon et al., 2007) and 12X v0 version (Adam-Blondon et al., 2011) were stored as SYNONYMS. SYNONYMS also include names and synonyms imported from the homologous *A. thaliana* and *O. sativa* gene entries using Inparanoid-based approach used previously for genome annotations of *F. vesca* (Shulaev et al., 2011) and *E. grandis* (Myburg et al., 2014). The FUNCTION values were extracted from the GO annotations derived from the IntrerproScan, Interpro2GO and EC2GO pipelines developed by Jaiswal laboratory that have been used for development of RiceCyc (Dharmawardhana et al., 2013) and MaizeCyc (Monaco et al., 2013).

#### Mapping of the reactions and pathways

After collecting the annotations for grape proteins in the ATTRIBUTE-VALUE standard format, and appropriately writing the GENETIC ELEMENT (required) and FASTA sequence files (optional), the “PathoLogic” option was executed (with taxonomic filtering on) to find best matches for grape proteins in the reference database MetaCyc. When a match was found for an entity, such as, an enzyme name, gene name, synonym, EC number, reaction etc., then the respective reaction and pathway were projected in the VitisCyc.

#### Quality control filters

To ensure consistency and accuracy, a quality control check was performed after initial assembly of VitisCyc, following the standard procedure of the Pathway Tools suite (Karp et al., 2002) with taxonomic filtering on. Subsequently, enrichment of plant pathways in the VitisCyc were done by comparing it with PlantCyc (version 5.0), a plant-specific metabolic network (Chae et al., 2012). If, different names for the same protein were found across various sources, then enzyme names were manually edited to match with associated EC and GO terms, while other names were included as FUNCTION-SYNONYM attributes for EC/GO match. The pathways specific to bacteria, fungi and animals that bypassed the taxonomic filtering were removed. In some cases, enrichment and manual curation of name-based assignments were also done. The PathoLogic tool provides a list of potential name matches and EC numbers (if available), along with the reactions. Often automated assignments fail due to change in the official enzyme names or due to assignment of new EC numbers, or no EC number for an enzyme. The annotations assigned due to partial or incomplete domain presence were also excluded. However, such errors are flagged alerting the curators to manually edit the list and make recommendations to confirm/edit/reject the existing reaction(s) or to create new reactions. Additional checks included visual confirmation of domains (InterPro) prior to final updates and rescoring of pathways. Pathway Tool software is updated routinely, whenever a new version becomes available, the current version is 18.0. Also, pathways and reactions are re-scored and crosschecked twice per year against the new release of the manually curated gold standard reference libraries provided by the MetaCyc and PlantCyc.

### Gene expression analyses using OMICs—Viewer tool

To show functionality of OMICs-Viewer tool, we used a subset of microarray expression data (NCBI GEO accession #GSE49569) from *Vitis vinifera* L. cv. Pinot noir clone Pommard (grafted to 101–14 rootstock, and trained in a double guyot system with vertically positioned shoots) described previously (Gouthu et al., 2014). In our study, we used expression data of 28,137 genes derived from pulp tissue of grape berries Collected at mid-véraison (when 50% of the berries in the cluster had changed color) representing four developmental stages: the prevéraison (PV) (before the ripening initiation), green-soft (GS), pink-soft (PS), and red-soft (RS). The ripening features of the berries are summarized in Supplementary Table 1.

In this data, 186 NimbleGeneIDs lacked mapping to 12X v1 geneIDs, and in 13 instances two NimbleGeneIDs mapped to single 12X v1 geneID. These inconsistencies are a result of differences between the the genome annotation used for desigining the NimbleGen expression array and 12X v1 genome annotation. The 186 irrelavent older gene models were excluded and in case of the 13 duplicate instances, we chose one NimbleGeneIDs out of two per gene for further analysis, based on its highest differential expression observed across the four developmental stages. We averaged the expression values from three replicates. Moderated paired *t*-tests were used to compare the mean log expression intensities between the berry classes; GS compared with PV, PS compared with GS, RS compared with PS and RS compared with GS. The R/Bioconductor Limma package (Benjamini and Hochberg, 1995; Smyth, 2004) was used to calculate fold change in the transcript level of a given gene between two stages as described previously (Gouthu et al., 2014). This non-redundant dataset (see Supplementary Table 2), representing the fold change in the expression of 27,949 genes during developmental transitions of grape berry, was uploaded and analyzed using OMICs-Viewer tool as described previously (Paley and Karp, 2006; Dharmawardhana et al., 2013; Monaco et al., 2013).

## Results

### VitisCyc as a resource for plant biologists

**Figure 1:**
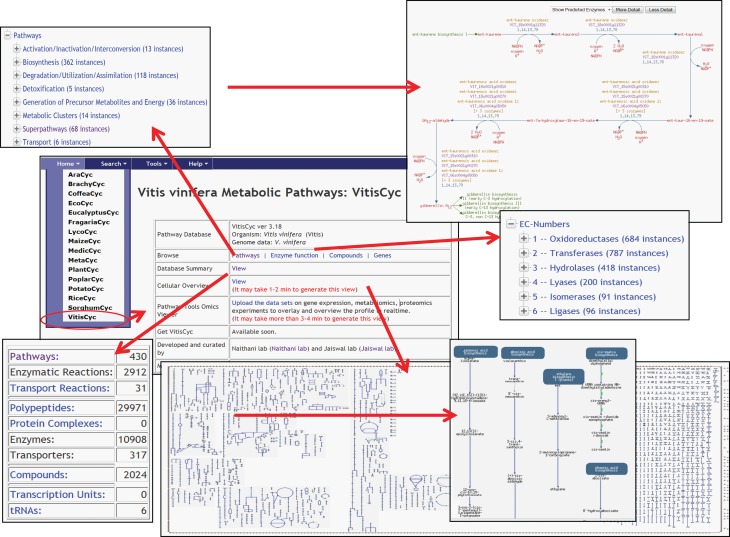
**A view of VitisCyc home page listing examples of various functions and utilities; a brief database summary, browsing of enzymes, and pathways using ontology based hierarchical classification scheme, view of detailed pathway page and cellular overview diagram**.

**Figure 2:**
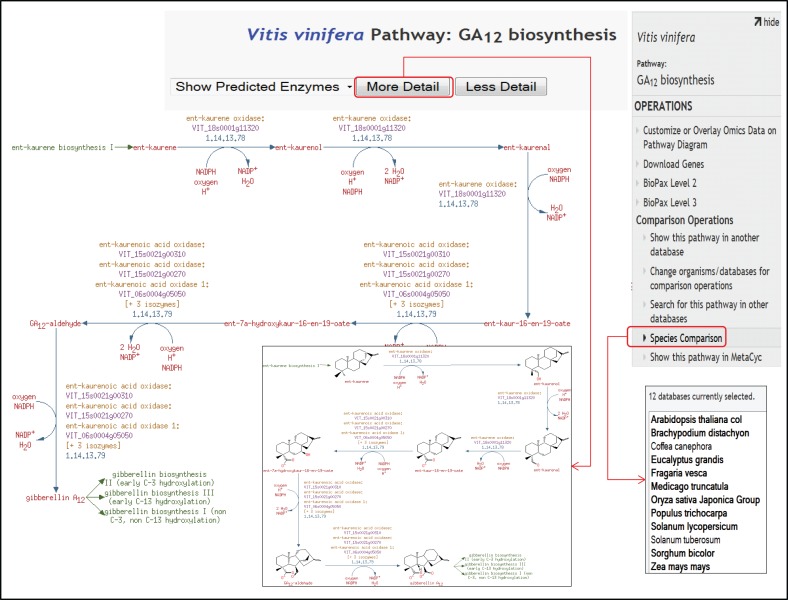
**A view of detailed pathway page showing GA12 biosynthesis pathway**. The pathway page provides three layers of granularity including the most detailed format displaying structures of metabolites and biochemical. The GA12 biosynthesis pathway also shows links highlighted in green color for the preceding ent-kaurene biosynthesis pathway I, as well as for the successive gibberellin biosynthesis pathway I and III that use GA12 as substrate. On the right side, species comparison icon is highlighted that allows users to make comparisons of pathways across other publicly available, species-specific plant pathway databases; AraCyc (Zhang et al., 2005), MaizeCyc (Monaco et al., 2013), MedicCyc (Urbanczyk-Wochniak and Sumner, 2007), PoplarCyc (Zhang et al., 2010), RiceCyc (Dharmawardhana et al., 2013), LycoCyc, CoffeaCyc, PotatoCyc, BrachyCyc, SorghumCyc, FragariaCyc (Shulaev et al., 2011), and EucalyptusCyc, accessible from the website http://pathways.cgrb.oregonstate.edu.

Currently, VitisCyc harbors 68 super pathways, 362 biosynthesis pathways, 118 catabolic pathways, 5 detoxification pathways, 36 energy related pathways and 6 transport pathways, 10,908 enzymes, 2912 enzymatic reactions, 31 transport reactions and 2024 compounds. Users can access VitisCyc online from the webcite http://pathways.cgrb.oregonstate.edu. The VitisCyc entry page provides a summary of the database, serves as a springboard to facilitate search and browsing, and provides links to a cellular overview diagram, and the OMICs-Viewer tool (Figure 1). Searches can be carried out using the “Search” link in the menu on the top of the page, which may require selecting the species “*Vitis vinifera.”* The browsing of the genes, enzymes, compounds, and pathways are supported using a framework of ontology based hierarchical classification schema. By using the hyperlinks, users can navigate up to the detailed pathway pages and views associated with each metabolite, gene ID, enzyme activity, reaction, and literature citations (Figure 2). The pathway page allows users to customize the levels of details shown on the page. The most detailed pathway view displays the structures of the metabolites. Whenever applicable, in the pathway page experimentally verified enzymatic activities or gene functions are depicted in bold. By clicking on the species comparison icon (Figure 2, on right side) users can compare a given pathway with reference databases, MetaCyc (Caspi et al., 2014), PlantCyc (Chae et al., 2012), and EcoCyc (Karp and Caspi, 2011), and/or across two or more species-specific, plant pathways databases (Figure 2) accessible from http://pathways.cgrb.oregonstate.edu. An example of detailed gene page is shown in Supplementary Figure 1. For extended help, users are encouraged to view online video tutorials on Cyc databases available at http://biocyc.org/webinar.shtml.

### Manual curation of VitisCyc

As described in the Methods, we projected a grape metabolic network based on extensive computational analyses, quality control steps, and manual curation. Manual curation includes (i) confirmation of computational mappings of genes to reactions and pathways, (ii) addition of grapevine-specific pathways, reactions, metabolites and biochemicals, and (iii) editing of reactions, sub-pathways, pathways and super-pathways including assignment of genes to a given enzyme and relevant pathways based on published grape literature. Examples of the manually curated pathways include four pathways of gibberellins and gibberellin precursors biosynthesis (Mattivi et al., 2006; He et al., 2010; Giacomelli et al., 2013); ten pathways involved in biosynthesis of five experimentally identified anthocyanins (cyanidin, delphinidin, peonidin, petunidin, malvidin and pelargonidin), and their 3-O-monoglycosides, and acyl-glycosides derivatives (Mattivi et al., 2006; He et al., 2010). We have also curated biosynthesis pathways and all associated entities for experimentally known flavonols in grapes, such as myricetin, quercetin, laricitrin, kaempferol, isorhamnetin and syringentin (Mattivi et al., 2006). Changes were also made in the biosynthesis pathways of plant hormones (e.g., abscisic acid, ethylene biosynthesis, jasmonic acid); trans-lycopene; pro-anthocyanidins; and isoflavonoids. In the generic interest of grape research community, we will continue to host pathways that can be useful in analyzing the data from other *Vitis* species, even though experimental evidence suggest their absence in *Vitis vinifera*.

Based on the published literature from *V. vinifera*, we entered 50 compounds, created 70 reactions, ~20 sub-pathways, assigned or edited function of ~150 genes associated with 80 reactions and deleted 10 subpathways. We also added 82 grape-specific literature references. Out of ~2024 metabolic compounds and biochemicals in VitisCyc, we have verified existence of about ~400 (20%) entities by mining the published literature.

### Upload, display and analysis of user-defined expression data

**Figure 3:**
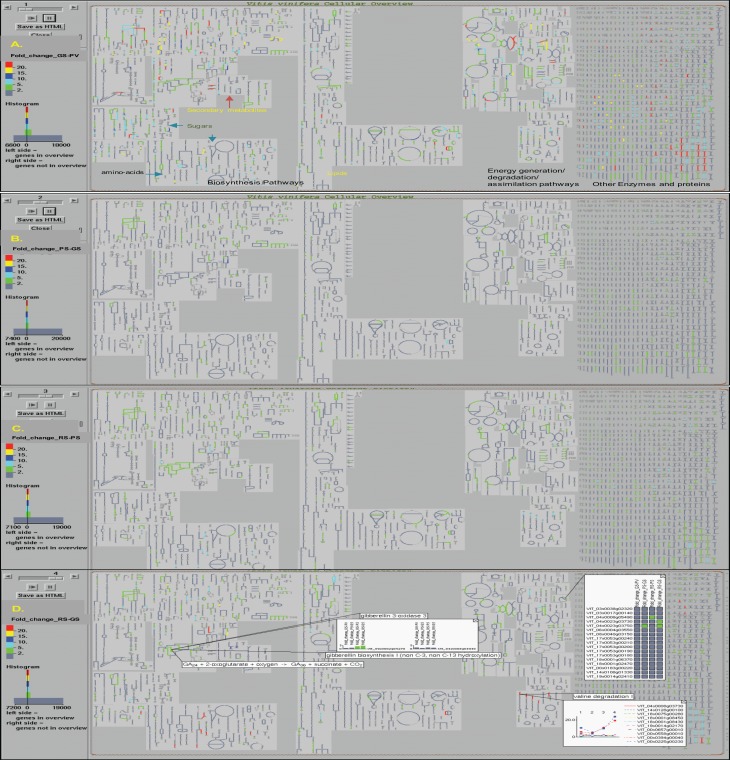
**The *Vitis vinifera* cellular overview diagram overlaid with expression data shows changes in the overall cellular gene expression profile in the pulp tissue of grape berry during ripening transitions observed in **(A)** green soft (GS) stage in comparison to prevéraison (PV) stage; **(B)** pink soft (PS) stage in comparison to GS stage; **(C)** red soft (RS) stage in comparison to PS stage; and **(D)** RS stage in comparison to early GS stage**. The change in the gene expression values during ripening transitions are depicted by colors, red (>20 fold), yellow (15–20 fold), dark blue (10–15 fold), light blue (5–10 fold) and green (2–5 fold). The examples of information accessible to users from the interactive cellular overview platform on reactions, pathways, compounds, and expression profiles of genes are shown **(D)**. More details can be accessed by clicking on any entity and data can be displayed on pathways.

The OMICs-Viewer tool, available from the VitisCyc entry page (Figure 1) or from the “Tools” menu available from the top navigation bar, enables users to upload and analyze a wide array of expression data including transcriptomes, proteomes, metabolomes, reaction flux, and/or any other data that can be assigned to metabolic genes, proteins, compounds, or reactions. The microaaray expression data (Supplementary Table 2) representing fold change in the expression of 27,949 unique genes in the pulp tissue of grape berries during four developmental transitions; in GS stage compared to PV; in PS stage compared to GS; in RS stage compared to PS, and in RS stage compared to GS was uploaded on OMICs-Viewer. We were able to map 7,566 out of total 27,949 genes to VitisCyc metabolic network. The remaining genes encode for transcription factors, regulatory proteins, structural proteins and for proteins of unknown function which are not yet associated to any metabolic reactions or pathways. Figure 3 shows relative change in the expression of grape genes during ripening transitions. The red color lines represent highest change in expression followed by yellow, dark blue, light blue and green in the cellular overview diagram.

We observed major changes at the cellular transcriptome during PV to GS transition that affects a wide array of genes associated with both primary and secondary metabolisms, hormone metabolism, photosynthesis, and energy pathways (Figure 3A). In contrast, the subsequent developmental transition from GS to PS mostly affects genes involved in secondary metabolic pathways (Figure 3B). During PS to RS transition, the change in the expression of genes of primary metabolic pathways and energy pathways can be observed in spite of major changes occurring in the plant hormone and secondary metabolic pathways (Figure 3C). The change in the expression of genes in the RS stage compared to GS is shown in Figure 3D.

**Figure 4:**
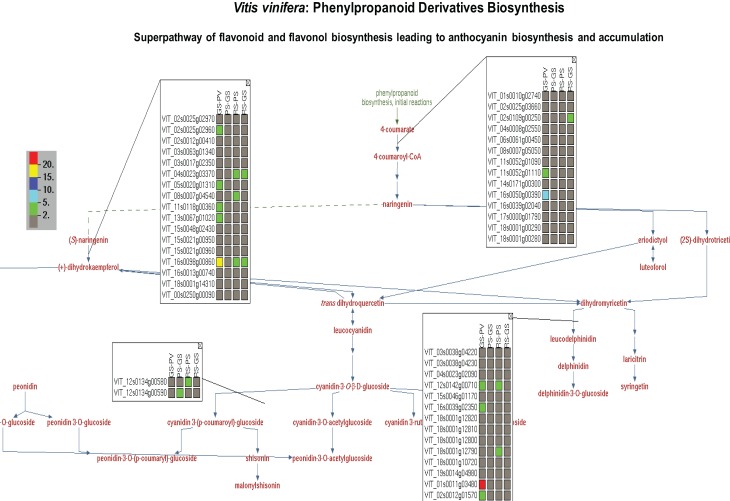
**A view of superpathway of flavonoid and flavonol biosynthesis leading to anthocyanin biosynthesis and accumulation showing expression profile of genes associated with individual reactions**. The genes expression data depicts change in the expression of individual genes in the pulp tissue of grape berry during four ripening transitions. GS-PV: fold change in gene expression in green soft stage compared to prevéraison stage. PS-GS: fold change in gene expression in pink soft stage compared to green soft stage. RS-PS: fold change in gene expression in red soft, ripe stage compared to pink soft stage. The fold change in the gene expression values are depicted by red (>20), yellow (15–20), dark blue (10–15), light blue (5–10) and green (2–5) colors. By clicking on “More Detail” icon users can see functional annotations of the genes and chemical structure of the compounds.

Taking advantage of the OMICs-Viewer's interactive environment, we compiled a list of differentially expressed genes coding for enzymes of flavonol, flavonoid, anthocyanin, terpene, polysaccharides and plant hormone pathways (Supplementary Table 3). Clicking on a genes or a reaction on cellular overview diagram allowed us to survey various pathway pages. Figure 4 displays minimal view of the superpathway of flavonol, flavonoid, anthocyanins biosynthesis with the expression profile of few genes. By clicking on the “More Detail” icon on this pathway page, additional information, such as enzyme names with EC numbers and gene annotations can be displayed (see Supplementary Figure 2). A quick glance at the list and detailed pathway pages show that gene duplicates or members of a gene family carrying the same functional annotations, and thus mapped to the same reaction, do not share identical expression profiles (Figure 4, Supplementary Table 3). The change in the gene expression observed here represents only pulp tissue. It is likely that dramatic changes in the expression profile of the genes of this particular pathway could be observed in the skin or seed of grapes during the ripening transition as suggested by previous studies (Davies et al., 2010).

A majority of genes associated with various pathways (Supplementary Table 3) show differential expression during one or more ripening stage transitions, although transcription of the individual genes may peak either at early or late stage. Phytohormones control a variety of developmental processes in plants including fruit development and ripening (White, 2002). Therefore, we surveyed changes in the transcription of genes associated with the plant hormones metabolism (Supplementary Table 3). We found that 10 out of 13 genes of ethylene biosynthesis pathway show increased expression during PV to GS transition (ranging from 2 to ~15 fold). During GS to PS transition, the transcript's level of 9 out of 13 genes slightly decline, while the transcript's level of 4 genes remain more or less stable. In contrast, during PS to RS transition 9 out of 13 genes show increase and 4 genes show slight decline in transcription. These observations are consistent with the previous reports showing rise in the ethylene level before the ripening onset (Chervin et al., 2004; Sun et al., 2010) and increase in the transcripts level of ethylene biosynthesis genes before véraison (Ziliotto et al., 2012; Bottcher et al., 2013). Abscisic Acid is considered as the ripening promoter of grape berry and transiently increases during véraison (Deluc et al., 2009; Wheeler et al., 2009). We observed that 8 genes associated with Abscisic acid biosynthesis are upregulated (2 to ~86 fold-change) during PV to GS transition and then slightly decline in the subsequent stages except *VIT_02s0087g00930* coding for 9-cis-epoxycarotenoid dioxygenase 2 (Supplementary Table 3). Incidentally, the three genes encoding for ABA 8'-hydroxylase, involved in abscisic acid breakdown, exhibit sharp decline in transcription during PV to GS transition and continue to decline with ripening progress (Supplementary Table 3). Interestingly, 17 out of 30 genes involved in jasmonic acid biosynthesis show 2–5 folds increase, while 13 biosynthesis genes show decline in transcription during PV to GS transition (Supplementary Table 3). However, in subsequent ripening transitions, the transcript levels of the majority of jasmonic acid biosynthesis genes either show decline or remain stable. The function of gibberellin in berry development is mostly confined to the early stages of fruit formation and the berries do not maintain high levels of gibberellic acids (GAs) during the ripening (Giacomelli et al., 2013). The transcripts levels of most genes associated with gibberellin biosynthesis pathways decline during the berry ripening (Supplementary Table 3). Similarly, brassinosteroids are known to promote accumulation of pigments in grape (Symons et al., 2006). All three differentially expressed brassinosteroid biosynthesis genes show decrease in transcription during ripening.

Overall, the down-regulation of gibberellin and brassinosteroid biosynthesis related transcripts and the up-regulation of ethylene and ABA biosynthesis related transcripts suggest coordinated action of these hormones during the onset of grape berry ripening.

## Discussion

VitisCyc aims to provide a comprehensive view of grapevine-specific cellular metabolic networks, and a framework for analysis of systems level OMICs expression data. VitisCyc was constructed using Pathway Tools software (Karp et al., 2002) by integrating publicly available knowledge of grape metabolism with an *in silico* metabolic networks model based on the *Vitis vinifera* sequenced genome. The grape genome was sequenced first at 8X coverage (Jaillon et al., 2007), that was followed by release of genome annotation versions 12X v0 (Adam-Blondon et al., 2011) and 12X v1(Grimplet et al., 2012). Thus, users struggle with three genome annotations, back and forth to relate their findings with published studies, and publicly available data. In VitisCyc, we use the most updated 12X v1 annotations available from CRIBI (http://genomes.cribi.unipd.it/grape/), and also provide the gene annotation from 8X and 12X v0 annotations, if the gene model has not changed (Supplementary Figure 1). In the future, we would be able to incorporate new versions of genome annotation data as and when it becomes available. Our ability to map different geneIDs for existing gene models with incremental updates to gene annotations, pathways, reactions, and compounds (small molecules) allows users to build correspondence with the previously published research more easily.

The OMICs-Viewer, a built in tool (Paley and Karp, 2006) within VitisCyc, allows researchers to upload data of their choice, to paint it over the cellular overview diagram, and analyze expression of genes in context of a pathway and overall cellular metabolic network. Previously, we have shown analyses of microarray expression data and RNA-Seq data using the OMICs-Viewer tool in RiceCyc (Dharmawardhana et al., 2013) and MaizeCyc (Monaco et al., 2013), respectively. In this study, we used a subset of data, generated by Gouthu et al. (2014) that comprises gene expression data from pulp tissue of grape berries of four ripening stages: Green Hard, prevéraison stage (PV), Green Soft (GS), Pink Soft (PS), and Red Soft (RS). We demonstrated how information linked to a compound, reaction and genes can be accessed (Figure 3D) from the cellular overview diagram, and gene expression profiles can be painted on the pathway page. Users can navigate back and forth between the cellular overview diagram and the detailed pathway pages to pick differentially expressed pathways and genes. In addition to examples shown here, users can define maximum cut-off values, choose zero-centered or 1-centered scale to extract desired information, generate a table of individual pathways and associated genes exceeding the defined threshold, and/or choose to display data values on the genomic map. Data from multiple treatments/samples can also be uploaded simultaneously to generate animations of cellular expression profiles across samples.

Gouthu et al. (2014) have previously identified ~3000 differentially expressed genes by analyzing the skin, pulp, and seed tissues from Green Hard prevéraison (PV), Green Soft (GS), Pink Soft (PS), and Red Soft (RS) grape berries collected from mid-véraison cluster (when color change was visible in 50% of the berries). Their study was focused on asynchronous ripening between grape berries within the same cluster and suggested that in spite of the difference in the pace of ripening, the overall transcriptional program is similar between early and late ripening berries (Gouthu et al., 2014). Using OMICs-Viewer tool, we are able to extract additional information that was not garnered in the previous study. Our analysis suggests that the PV to GS transition triggers major changes in the overall cellular transcription activity leading to berry ripening and changes in the cellular metabolic profile (Figure 3). Mapping expression data on pathways allowed us not only to identify key reactions within various metabolic pathways that are up- and/or down regulated during ripening transitions, but also to account for the specific contribution of individual homologs mapped to the same reaction (Supplementary Table 3). Plant genomes harbor extensive gene duplications and large gene families (Shiu and Bleecker, 2003; Jaillon et al., 2007; Velasco et al., 2007; Myburg et al., 2014; Renny-Byfield and Wendel, 2014). The duplicate genes or members of a gene family carry the same functional annotations and thus map to the same reactions in the network unless an alternate function has been documented. Our analysis showed that often gene-duplicates do not share identical expression profiles and thus, are likely to be under the control of different regulators. Such information is specially useful in identification of candidate genes that are expressed in a specific cell- or tissue-type, at a particular stage of development, and in response to the changes in the plant's immediate environment. Users can also identify genes with redundant function and compare overlaps in expression profiles of genes associated with different reactions of a given pathway.

In addition, our analysis shows interplay between several hormones that is required to enable the fruit to transition from berry maturation to the ripening phase (Mcatee et al., 2013; Osorio et al., 2013), which is especially important to understand ripening process in non-climacteric fruits, including grape. We obsereved decline of gibberellins and brassinosteroids and up-regulation of ABA and ethylene during the ripening transitions, especially at GS compared to PV (Supplementary Table 3). Ethylene is known as a major ripening regulator in climacteric fruits, but it is not considered a major regulator in non-climacteric fruits. However, our data identified that 75% of ethylene biosynthesis genes were up-regulated during PV to GS transition and therefore, supports role of ethylene in grape berry ripening, which is recently emerging (Ziliotto et al., 2012; Bottcher et al., 2013). Our data suggests that jasmonic acid is likely to play a role in the early onset of grape berry ripening (Supplementary Table 3). Knowledge about the role of jasmonic acid in grape berry is slowly emerging (Kondo and Fukuda, 2001; Fortes et al., 2011) and recent reports suggest its involvement in the ripening of another non-climacteric fruit strawberry (Concha et al., 2013; Preuss et al., 2014), and also in ethylene independent induction of lycopene biosynthesis in tomato (Liu et al., 2012). Overall, the finely tuned sampling of ripening stages, the extensive manual curation of the hormone-related genes performed by our group, and the data visualization capacity of the OMICs-Viewer tools provide a clearer picture of the action of these hormones in the context of the grape metabolic network.

We view publicly available experimental data as a vital resource for improvement of the functional annotation of the grapevine genes and curation of enzymes, reactions, pathways, and compounds in the VitisCyc databases. Manual curation is an ongoing process and the growth of VitisCyc is expected as more data becomes available and integrated. Many grape researchers rely on another publicly available grape database VitisNet (Grimplet et al., 2012) and MapMan (Carbonell-Bejerano et al., 2013) for expression data analysis. VitisNet was built with CellDesigner platform and allows users to download one or more molecular networks from VitisNet and analyze their data using Cytoscape (Grimplet et al., 2009, 2012). We will be looking for opportunities to coordinate curation efforts especially on signaling pathways and small molecules together with the resources like MapMan (Ramsak et al., 2014), VitisNet and Plant Reactome (Monaco et al., 2013). We will also focus on efforts for developing automated scripts capable of extracting information from research articles with increased precision in order to provide more adequate information needed for OMICs data analysis, and the descriptions associated with biological function of genes/enzymes. We envision that in future, we could also provide a link to the phenotype database and to a metabolic mutant phenotype.

We expect that VitisCyc will aid grape researchers in discovering the broader role of new and known genes and/or molecular interactions in context of plant development, cells, tissues and organ differentiation. It could help in understanding how a plant's environment and genetic diversity relate to yields and the quality of its fruit's taste, aroma, flavor, and texture, and how the plant's overall metabolism and its ability to adapt is affected in response to climate change.

The VitisCyc is freely accessible online at http://pathways.cgrb.oregonstate.edu. The data content in the VitisCyc will be maintained and updated twice a year by continuously surveying the public resources and research articles as long as we have some support for curation and afterwards an archive copy will be maintained.

### Conflict of interest statement

The Guest Associate Editor Mario Pezzotti declares that, despite having collaborated with author Laurent G. Deluc, the review process was handled objectively and no conflict of interest exists. The author Laurent G. Deluc declares that, despite having collaborated with the editor Mario Pezzotti, the review process was handled objectively and no conflict of interest exists. The authors declare that the research was conducted in the absence of any commercial or financial relationships that could be construed as a potential conflict of interest.
