# Supplementary material for: VitisCyc: a metabolic pathway knowledgebase for grapevine (Vitis vinifera)
Source: Front Plant Sci. 2014 Dec 9;5:644. doi: 10.3389/fpls.2014.00644 (PMC4260676; doi:10.3389/fpls.2014.00644)
Supplement: Supplementary file 1 [file Table1.PDF]

Supplementary Table 1: Four developmental stages of grape berry used for gene expression studies and their sugar and pigment accumulation levels.

Ripening stages	PV	GS	PS	RS
Sugar level (°brix)	4.3± 0.1	8.3± 0.2	10.1± 0.3	12.6± 0.2
Color Index (CI)	0.6± 0.05	0.8± 0.05	2.2± 0.13	3.3± 0.14

PV: pre-véraison; GS: Green Soft; PS: Pink Soft; RS: Red Soft. Sugar levels were measured as °brix using refractometer (SPER Scientific, USA) and the color indices were computed using a CR310 Chromameter as described earlier by Gouthu et al. (2014).
