# Supplementary figures and images for: VitisCyc: a metabolic pathway knowledgebase for grapevine (Vitis vinifera)
Source: Front Plant Sci. 2014 Dec 9;5:644. doi: 10.3389/fpls.2014.00644 (PMC4260676; doi:10.3389/fpls.2014.00644)

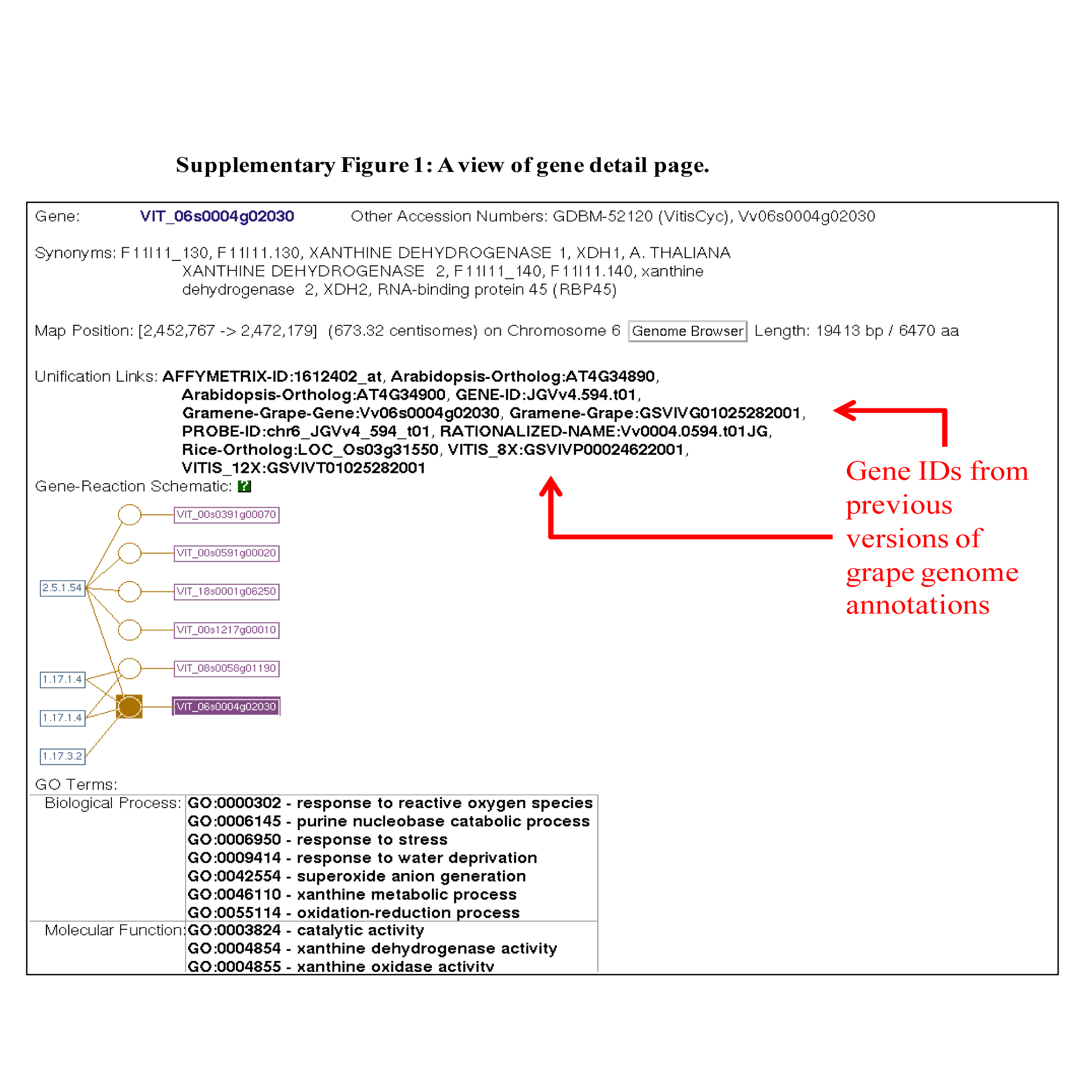

Supplement: Supplementary file 4 [file Image1.JPEG]
